# Multiplicity of enzymatic functions in the CAZy AA3 family

**DOI:** 10.1007/s00253-018-8784-0

**Published:** 2018-02-06

**Authors:** Leander Sützl, Christophe V. F. P. Laurent, Annabelle T. Abrera, Georg Schütz, Roland Ludwig, Dietmar Haltrich

**Affiliations:** 1 Food Biotechnology Laboratory, Department of Food Science and Technology, BOKU—University of Natural Resources and Life Sciences Vienna, Muthgasse 11, A-1190 Wien, Austria; 2 Doctoral Programme BioToP—Biomolecular Technology of Proteins, BOKU—University of Natural Resources and Life Sciences Vienna, Muthgasse 18, A-1190 Wien, Austria; 3 University of the Philippines Los Baños, College Laguna, Los Baños, Philippines

**Keywords:** Cellobiose dehydrogenase, Glucose oxidase, Aryl-alcohol oxidase, Methanol oxidase, Pyranose oxidase, Glucose dehydrogenase, Lignocellulose degradation

## Abstract

The CAZy auxiliary activity family 3 (AA3) comprises enzymes from the glucose-methanol-choline (GMC) family of oxidoreductases, which assist the activity of other AA family enzymes via their reaction products or support the action of glycoside hydrolases in lignocellulose degradation. The AA3 family is further divided into four subfamilies, which include cellobiose dehydrogenase, glucose oxidoreductases, aryl-alcohol oxidase, alcohol (methanol) oxidase, and pyranose oxidoreductases. These different enzymes catalyze a wide variety of redox reactions with respect to substrates and co-substrates. The common feature of AA3 family members is the formation of key metabolites such as H_2_O_2_ or hydroquinones, which are required by other AA enzymes. The multiplicity of enzymatic functions in the AA3 family is reflected by the multigenicity of AA3 genes in fungi, which also depends on their lifestyle. We provide an overview of the phylogenetic, molecular, and catalytic properties of AA3 enzymes and discuss their interactions with other carbohydrate-active enzymes.

## Introduction

The Carbohydrate-Active enZYme (CAZy) database (http://www.cazy.org/) describes families of structurally related catalytic modules and domains of enzymes that degrade, modify, or create glycosidic linkages. The classification system in this database is founded on amino acid sequence similarities, protein folds, and catalytic mechanisms. The catalytic modules or enzymes in this database are grouped in families of glycoside hydrolases (GH), polysaccharide lyases (PL), carbohydrate esterases (CE), and glycosyltransferases (GT); in addition, the non-catalytic carbohydrate-binding modules (CBM) associated with these catalytic modules are included in the database as well (Lombard et al. 2014).

It is now understood that the main chains of polysaccharides such as chitin, cellulose, or starch are not only cleaved by hydrolytic mechanisms but also by oxidative reactions catalyzed by lytic polysaccharide monooxygenases (LPMO; (Beeson et al. 2015)). Because of this and the intimate link between plant cell wall polysaccharides and lignin—and thus the necessity to degrade lignin as well to achieve efficient cell wall deconstruction—the curators of the CAZy database recently added a new enzyme class, termed “Auxiliary Activities” (AA), which comprises redox enzymes that act in conjunction with CAZymes. These auxiliary activities include for example the abovementioned LPMOs (families AA9, AA10, AA11, and AA13) as well as redox enzymes involved in lignin breakdown, with the latter comprising well-studied laccases (AA1) or lignin-active class-II peroxidases (AA2) (Levasseur et al. 2013). A large and varying group of auxiliary activities is represented by family AA3. All of its members belong to the glucose-methanol-choline (GMC) family of oxidoreductases (Cavener 1992) and depend on a flavin-adenine dinucleotide (FAD) cofactor for their activity. Some AA3 members are very well characterized both from enzymological and structural view; yet overall, this group is still functionally enigmatic, with possible functions of some of its members in, e.g., lignocellulose degradation emerging only slowly (Kracher et al. 2016).

**Fig. 1:**
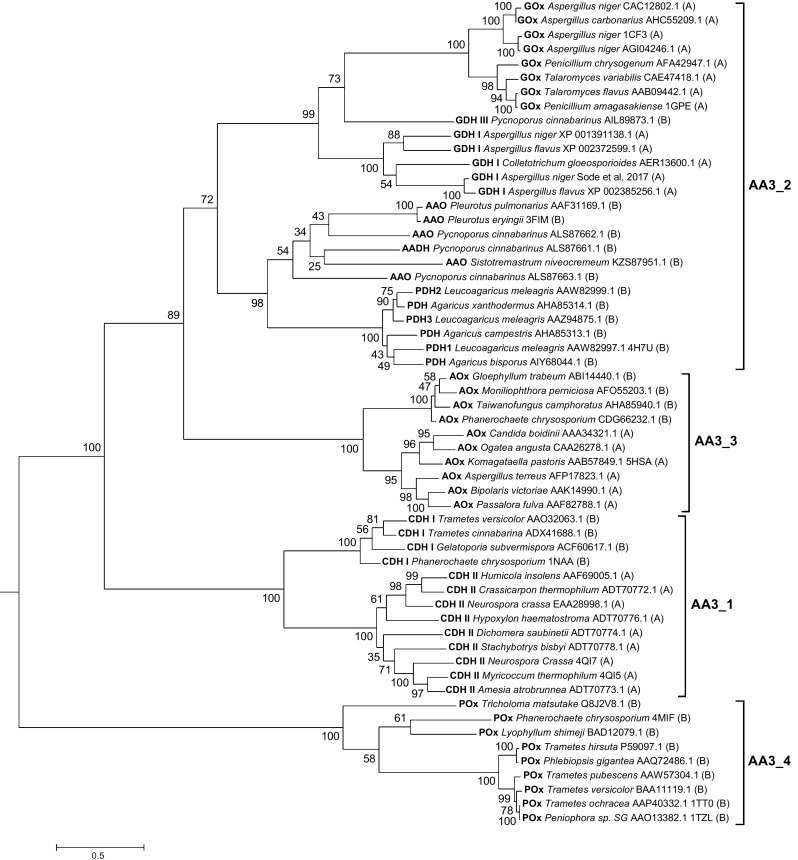
Phylogeny of the AA3 family of fungal GMC oxidoreductases. The phylogenetic tree was calculated using sequences of 58 experimentally characterized enzymes, aryl-alcohol oxidase (AAO), aryl-alcohol dehydrogenase (AADH), alcohol oxidase (AOx), cellobiose dehydrogenase flavodehydrogenase domain (CDH, class I + II), glucose dehydrogenase (GDH, class I + III), glucose oxidase (GOx), pyranose dehydrogenase (PDH), and pyranose oxidase (POx) of fungal origin belonging to the phylum of ascomycetes (**A**) or basidiomycetes (**B**). Sequence descriptions include species names, NCBI version numbers, and PDB codes if available. Sequences were aligned using M-coffee (Wallace et al. 2006) with default settings. Phylogeny was inferred using PhyML (Guindon et al. 2010) and the Whelan and Goldman (WAG) amino acid substitution model (Whelan and Goldman 2001). Branch support was calculated by 500 bootstrap repetitions (values displayed in percent). The tree was visualized in MEGA7 (Kumar et al. 2016) and rooted on midpoint

A comparison based on a phylogenetic inference using sequences of 58 biochemically characterized enzymes (Fig. 1) shows that family AA3 is further divided into four subfamilies: AA3_1 (including the flavodehydrogenase domains of cellobiose dehydrogenase, CDH), AA3_2 (aryl-alcohol oxidoreductases, both oxidases, AAO and dehydrogenases, AADH, and glucose 1-oxidases, GOx; here we also included glucose 1-dehydrogenases, GDH, and pyranose dehydrogenases, PDH, based on their high-sequence similarities to the former two enzymes), AA3_3 (alcohol oxidases; AOx), and AA3_4 (pyranose oxidases, POx).

**Fig. 2:**
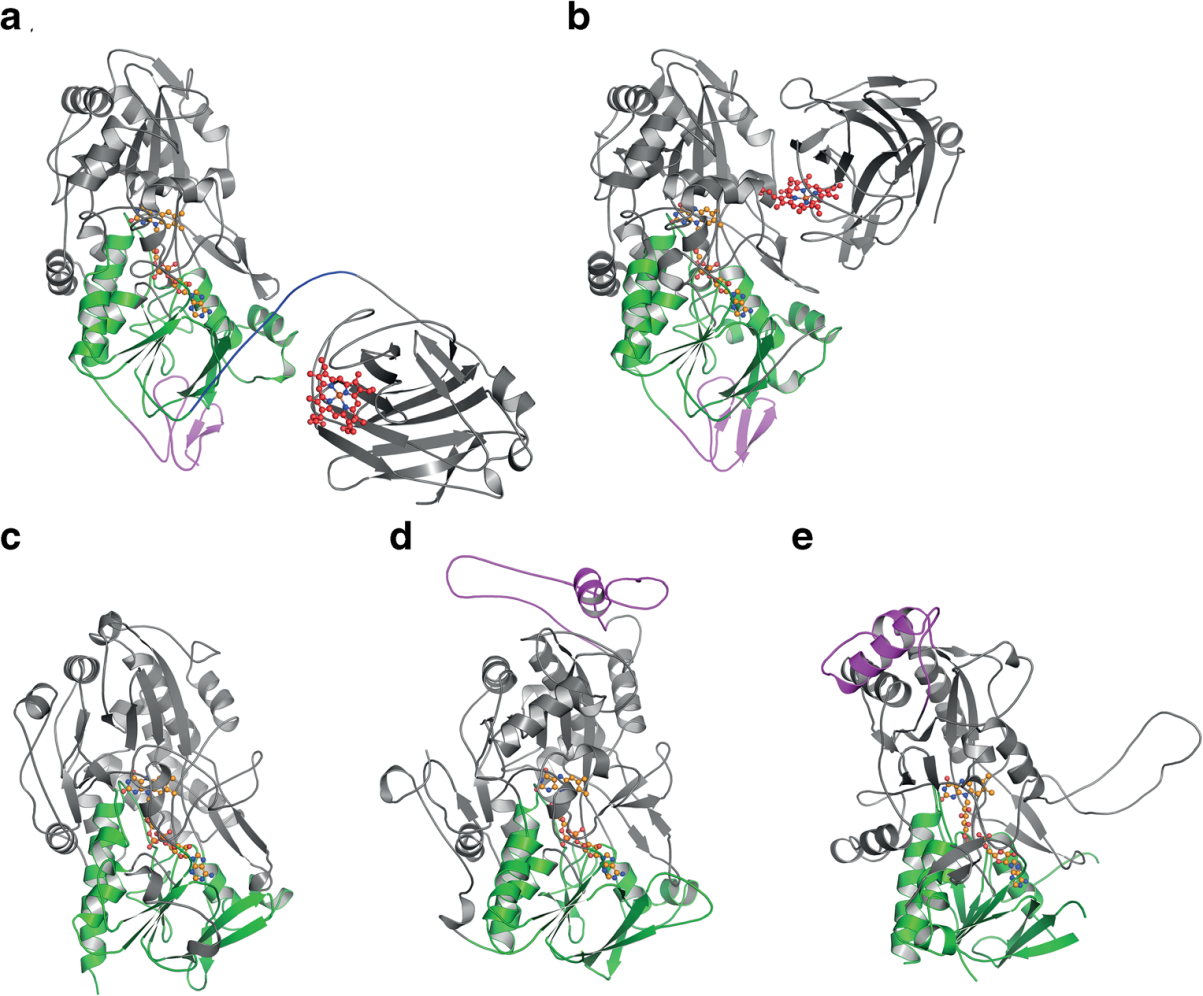
Molecular structures of representative members of the AA3 families in cartoon representation. **a** *Neurospora crassa* cellobiose dehydrogenase CDH IIA in the open conformation (PDB 4QI7 (Tan et al. 2015)), **b** *Myricoccum thermophilum* cellobiose dehydrogenase in the closed conformation (PDB 4QI6 (Tan et al. 2015)), **c** subunit of homo-dimeric *Aspergillus niger* glucose oxidase GOx1 (PDB 1CF3 (Wohlfahrt et al. 1999)), **d** subunit of homo-octameric *Pichia pastoris* alcohol oxidase AOx1 (PDB 5HSA (Koch et al. 2016)) and **e** subunit of homo-tetrameric *Phanerochaete chrysosporium* pyranose oxidase (PDB 4MIF (Hassan et al. 2013)). The FAD-binding domain is highlighted in green; FAD is shown in orange (ball-and-stick representation), and the haem *b* in the cytochrome domain of CDH is depicted in red (ball-and-stick representation). The carbohydrate-binding module (CBM1) of the two CDHs (**a**, **b**), the insertion loop of *Pp*AOx1 (**d**) and the head domain of *Pc*POx (**e**) are shown in magenta

A flavin-dependent enzyme of the GMC family is typical composed of a flavin-binding domain comprising the N-terminal, the C-terminal and an internal region of the sequence, and a substrate-binding domain comprising two internal, discontinuous regions of the sequence (Cavener 1992; Kiess et al. 1998). Some GMC family members may contain structurally distinct loops or even additional domains (Fig. 2). The flavin-binding domain is highly conserved in all members of the GMC family and shows the canonical Rossmann fold or *βαβ* mononucleotide-binding motif, interacting with the ADP moiety of FAD. The sequence and structure of the substrate-binding domain of GMC oxidoreductases are less preserved, which reflects the diversity in substrate specificity within this family. Even though the substrates (various carbohydrates or alcohols) oxidized by these GMC oxidoreductases are diverse, the overall reaction mechanism of the FAD-dependent enzymes is similar. The substrate oxidation involves a direct hydride transfer from the substrate to the N5 atom of the isoalloxazine moiety of FAD, resulting in reduced FADH_2_ (reductive half-reaction). FADH_2_ is subsequently re-oxidized (oxidative half-reaction) by either oxygen (resulting in the formation of hydrogen peroxide) or by alternative electron acceptors such as different quinones or (complexed) metal ions (resulting in the corresponding hydroquinones or reduced metal ions).

Putative genes encoding members of the AA3 family are predominantly found in fungal organisms, both ascomycetes and basidiomycetes, but they are also found in insects such as *Drosophila melanogaster* or *Bombyx mori* where they are thought to play a role in immunity and development (Iida et al. 2007; Sun et al. 2012). Often multiple genes of members of a certain subfamily are found in one species, albeit this multiplicity of genes shows considerable discrepancies among the different AA3 subfamilies as was shown by an analysis of 41 fungal genomes (Levasseur et al. 2013). Multigenicity is most pronounced in the AA3_2 subfamily, with for example 22 putative AA3_2 genes found in *Coprinopsis cinerea* (GOx and AAO only, (Levasseur et al. 2013), 26 putative AA3_2 genes in *Aspergillus niger* CBS 513.88, or a total of 31 AA3 genes in *Stereum hirsutum* (Kracher et al. 2016). Multigenicity is less pronounced in subfamilies AA3_3 and AA3_4, with up to seven putative AA3_3 genes in *Stereum hirsutum* or a maximum number of three AA3_4 genes in *Auricularia delicata*, again based on the comparison of 41 fungal genomes (Levasseur et al. 2013). Multigenicity is least pronounced in subfamily AA3_1. Genes of this subfamily are found in higher numbers in ascomycetes (averaging ~ 2.3 copies in 13 ascomycetes genomes), with a maximum number of four genes in *Botrytis cinerea*, whereas ~ 0.6 copies were detected in the genomes of 28 basidiomycete species, which typically showed only a single copy of this AA3_1 gene when present. It should be pointed out that the majority of these genes are putatively assigned, and that most of the respective gene products have not been studied biochemically, e.g., with resect to their actual substrates. According to the CAZy website (http://www.cazy.org/AA3_characterized.html, accessed on Dec. 3, 2017) only 37 AA3 member proteins have been characterized biochemically.

### Subfamily AA3_1—cellobiose dehydrogenase

The first subfamily of the AA3 family consists of cellobiose dehydrogenases (CDH; EC 1.1.99.18, cellobiose:acceptor 1-oxidoreductase) (Levasseur et al. 2013), which are to date the only known *extracellular* hemoflavoproteins (Zamocky et al. 2006). CDH was first discovered and described by Eriksson and co-workers while analyzing the secretomes of the white-rot fungi *Trametes versicolor* and *Phanerochaete chrysosporium* in the presence of cellulosic substrates (Westermark and Eriksson 1974).

The monomeric multi-domain glycoprotein CDH harbors a haem *b* and an FAD cofactor, which are found in the N-terminal cytochrome (CYT_CDH_) and the C-terminal dehydrogenase (DH_CDH_) domain, respectively (Tan et al. 2015; Zamocky et al. 2006). The CYT domain with its haem *b* has also been classified as a separate auxiliary activity in the CAZy database, AA8 of iron reductases (Levasseur et al. 2013). The two domains of CDH are connected by a papain-sensitive flexible linker of approx. 20–35 amino acids (average of 28 ± 7 amino acids deduced from 293 CDH sequences), which imparts significant mobility to the individual CDH domains and allows an open (Fig. 2a) and a closed (Fig. 2b) conformation of CDH (Henriksson et al. 1991; Tan et al. 2015). Structures of the separate domains of *P. chrysosporium* CDH (*Pc*CDH) were published in the early 2000s (CYT_CDH_: 1.9 Å, PDB 1D7C, (Hallberg et al. 2000); DH_CDH_: 1.5 Å, PDB 1KDG, (Hallberg et al. 2002)), while structures of intact CDH were published only recently (*Neurospora crassa* CDH IIA: 2.9 Å, PDB 4QI7; *Myriococcum thermophilum* CDH: 3.2 Å, PDB 4QI6), due to the difficulties in obtaining suitable crystals of the flexible proteins (Tan et al. 2015). Harada et al. recently confirmed the domain movement by using atomic force microscopy (Harada et al. 2017). This conformational change and the mobility of the two domains are of particular importance for the electron transfer chain described below.

**Fig. 3:**
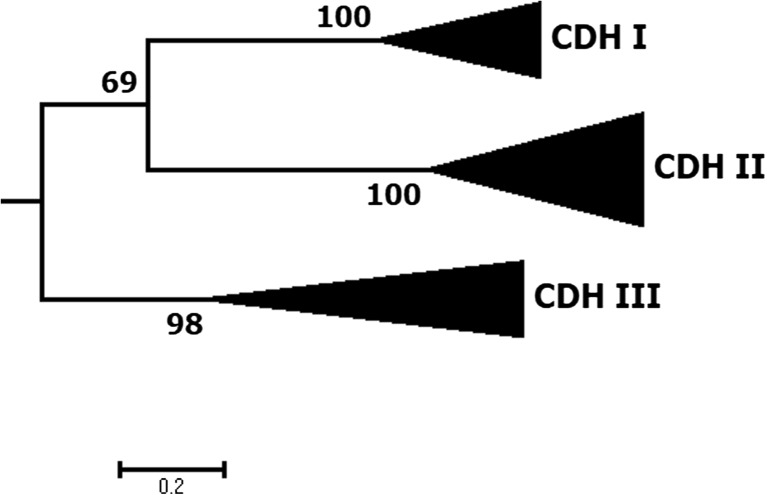
Phylogeny of cellobiose dehydrogenase (CDH); AA3_1 genes form three well-supported classes with distinct molecular and catalytic properties. The phylogenetic subtree was calculated using the flavin domains of 21 CDH sequences representative for CDH class I (6 sequences, 4 characterized), class II (9 sequences, 9 characterized), and class III (6 sequences, 0 characterized). The tree was rooted within AA3 family enzymes (not shown). Sequences were aligned using M-coffee (Wallace et al. 2006) with default settings. Phylogeny was inferred using PhyML (Guindon et al. 2010) and the Whelan and Goldman (WAG) amino acid substitution model (Whelan and Goldman 2001). Branch support was calculated by 100 bootstrap repetitions (values displayed in percent). The tree was visualized in MEGA7 (Kumar et al. 2016)

CDHs can be divided into three classes (Fig. 3), namely class-I, class-II, and class-III (Harreither et al. 2011; Zamocky et al. 2004, 2008). This division is based on phylogenetic analyses where basidiomycete CDHs are regrouped in class-I and ascomycete CDHs can be found both in class-II and class-III. Genes of the third class are found in ascomycetes and have yet to be characterized. CDHs of class-II can further be grouped into subclasses A and B, depending on whether CDH bears a C-terminal type-1 carbohydrate-binding module (CBM1), allowing the enzyme to bind firmly to cellulose or not. Class-I and class-IIB CDHs lack a CBM1; class-I CDHs can nevertheless strongly bind to cellulose by a yet unknown mechanism (Zamocky et al. 2006).

CDHs oxidize cellobiose, cellodextrins, or structurally related oligosaccharides such as lactose at C-1 of the reducing end. Cellobiose is thus oxidized to cellobionolactone (which then spontaneously hydrolyses to cellobionate) while the FAD cofactor is reduced to FADH_2_ (Hyde and Wood 1997). Other hemicellulose- and starch-derived oligosaccharides, such as xylo-, manno-, or malto-oligosaccharides are oxidized by a number of CDHs as well, albeit with lower catalytic efficiencies. CDHs typically discriminate monosaccharides and show lowcatalytic efficiency towards glucose, galactose, and mannose (Zamocky et al. 2006), for example, the specificity constant *k*_cat_·*K*_M_^−1^ of *Pc*CDH for cellobiose and glucose are 150 × 10^3^ M^−1^ s^−1^ and 0.015 × 10^3^ M^−1^ s^−1^, respectively (Kracher and Ludwig 2016). The carbohydrate substrate-binding site is located in the DH_CDH_ domain close to the isoalloxazine moiety of FAD, which is accessible through a 12-Å long tunnel (Hallberg et al. 2002). This binding site can also be accessed by the sugar substrates in the closed conformation of CDH (Tan et al. 2015). As a result of the reductive half-reaction, the two electrons that are taken up in the DH_CDH_ domain can subsequently be transferred to either a two-electron acceptor (e.g., 2,6-dichloro-indophenol (DCIP), which is routinely used in activity assays) or a one-electron acceptor (e.g., the haem *b* group in the CYT_CDH_ in an interdomain electron transfer) (Zamocky et al. 2006). Although this oxidative half-reaction can be achieved by a myriad of small molecular electron acceptors, it is the interdomain electron transfer from the DH_CDH_ FADH_2_ to the low redox potential CYT_CDH_ haem *b* that has been of particular interest over the past few years (Harada et al. 2017; Kadek et al. 2017; Kracher et al. 2016). In order to complete the electron transfer chain described above, the reduced haem *b* can in turn reduce a terminal electron acceptor such as copper-dependent LPMOs (Kracher et al. 2016; Phillips et al. 2011). Other electron acceptors of CDH that may be functionally important include variously (complexed) metal ions, most importantly Fe^3+^ or Mn^3+^ malonate (Ander 1994; Bao et al. 1993). CDH shows negligible activity with oxygen, even though it was originally termed cellobiose oxidase (Ayers et al. 1978). This reduction of metal ions at AA8 CYT_CDH_ could be involved, e.g., in the generation of highly reactive hydroxyl radicals (OH^**·**^) via Fenton’s reaction (see below). Interestingly, phylogenetically closely related AA8 domains were also found in a newly discovered group of fungal oxidoreductases that show a modular structure very similar to CDH, pyrroloquinoline quinone or PQQ-dependent pyranose dehydrogenases. PQQ-dependent PDH (which as a quinohaemoprotein is both structurally and biochemically different from FAD-dependent pyranose dehydrogenase of subfamily AA3_2) is classified in a separate family in CAZy, auxiliary activity AA12 (Matsumura et al. 2014). So far, PQQ-dependent PDH has only been described and characterized from the fungus *Coprinopsis cinerea* (Takeda et al. 2015).

### Subfamily AA3_2—aryl-alcohol oxidase/dehydrogenase

Aryl-alcohol oxidase (AAO; EC 1.1.3.7, aryl-alcohol:oxygen oxidoreductase) was first isolated and studied from different *Pleurotus* species (Bourbonnais and Paice 1988; Guillén et al. 1990; Sannia et al. 1991). AAOs are monomeric, two-domain enzymes containing non-covalently attached FAD, which are secreted by a number of wood-degrading fungi (Ferreira et al. 2015). The structure of AAO from *Pleurotus eryngii* (*Pe*AAO; 2.55 Å, PDB 3FIM, (Fernández et al. 2009)) shows a funnel-shaped channel connecting the active-site with the solvent. Access to this channel is limited by three aromatic residues, which also interact both with the (hydrophobic) alcohol substrate and oxygen during access to the buried FAD group.

AAOs typically catalyze the oxidation of a primary alcohol group of a range of different aromatic and aliphatic unsaturated alcohols, many of which are either secreted by fungi or formed during fungal decomposition of lignocellulose, to the corresponding aldehydes. Concomitantly, oxygen is reduced to hydrogen peroxide. *Pe*AAO is the best-studied AAO enzyme to date, both with respect to its structural and biochemical properties as well as its reaction mechanism (Hernandez-Ortega et al. 2012). *Pe*AAO shows high activity and a highcatalytic efficiency of 5.23 × 10^6^ M^−1^ s^−1^ with *p*-anisyl alcohol. Other alcohols that serve as good electron donor substrates for *Pe*AAO include veratryl alcohol, cinnamyl alcohol, or 2,4-hexadien-1-ol (Ferreira et al. 2006; Guillén et al. 1992).

A recent study of the *Pycnoporus cinnabarinus* genome together with secretome studies revealed four AA3_2 enzymes that are secreted during biomass degradation (Levasseur et al. 2014). One of these was identified as a glucose dehydrogenase; the other three showed high-sequence identities of 44.5–48.7% to *Pe*AAO (Mathieu et al. 2016). The corresponding proteins were recombinantly produced in *A. niger* and biochemically characterized. They showed comparable activities to AAOs for their reductive half-reaction in oxidizing a range of aromatic alcohols with catalytic efficiencies for, e.g., *p*-anisyl alcohol ranging from 0.631 × 10^3^ M^−1^ s^−1^ to 16.9 × 10^3^ M^−1^ s^−1^. *p*-Anisyl alcohol was also one of the preferred substrates of these three enzymes. The three isoforms, however, also differ significantly with respect to the reactivity with some of their electron donor substrates. *p*-Anisyl alcohol, for example, is oxidized by all three isoforms, where *m*-anisyl alcohol is only accepted by one of them with the other two showing negligible activity. While these three enzymes resemble AAO with respect to their alcohol substrates, one of these three enzymes did not show any activity with oxygen, and oxygen reactivity of the other two enzymes was low compared to the activity with alternative, quinoid electron acceptors such as *p*-benzoquinone or DCIP with relative dehydrogenase to oxidase activities of approx. 50:1 (Mathieu et al. 2016). Catalytic efficiencies for some of these electron acceptors were also very high, e.g., the enzyme termed *Pc*AAQO1 showed a *k*_cat_·*K*_M_^−1^ value of 0.877 × 10^6^ M^−1^ s^−1^ for the substrate *p*-benzoquinone (3-chlor-*p*-anisyl alcohol in saturating concentrations). In addition, these enzymes reduced phenoxy radicals that are formed by laccases during activity on lignin. Hence, these enzymes are no true oxidases but dehydrogenases, and they were termed aryl-alcohol quinone oxidoreductases (AAQO). It should be noted that the three *P. cinnabarinus aaqo* genes clustered with (biochemically characterized) AAO in a phylogenetic comparison (Mathieu et al. 2016). As with the electron donor substrates, reactivity of these AAQO isoforms of *P. cinnabarinus* differed considerably for different electron acceptor substrates as indicated by the catalytic efficiencies determined. The in vivo function of AAO had been suggested as a source of hydrogen peroxide for fungal peroxidases active on lignin. The occurrence of extracellular AAO-like enzymes that do not react with oxygen, or only very poorly do so, implicates that their role could go beyond the provision of H_2_O_2_.

### Subfamily AA3_2—glucose oxidase and glucose dehydrogenase

**Fig. 4:**
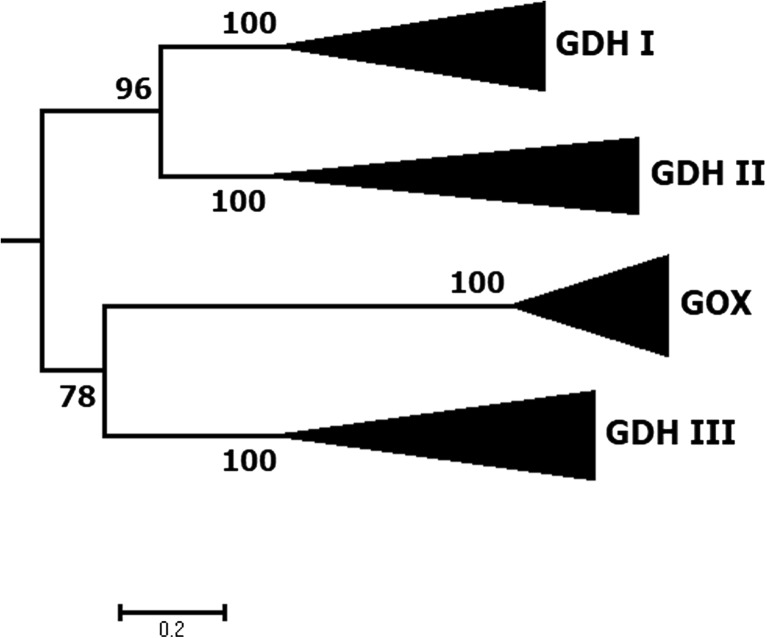
Detailed phylogeny of glucose oxidases and glucose dehydrogenases showing the newly proposed classification. An extended BLAST search resulted in numerous putative glucose oxidoreductases. The tree shown here was calculated using 28 GOx and GDH sequences representative for the clades GOx (8 sequences, 8 characterized), GDH class-I (7 sequences, 5 characterized), GDH class-II (6 sequences, 0 characterized), and GDH class-III (7 sequences, 1 characterized). The tree was rooted within AA3 family enzymes (not shown). Sequences were aligned using M-coffee (Wallace et al. 2006) with default settings. Phylogeny was inferred using PhyML (Guindon et al. 2010) and the Whelan and Goldman (WAG) amino acid substitution model (Whelan and Goldman 2001). Branch support was calculated by 100 bootstrap repetitions (values displayed in percent). The tree was visualized in MEGA 7 (Kumar et al. 2016)

Two different FAD-dependent enzymes are found in AA3_2, which specifically oxidize β-D-glucose (Glc) at the anomeric carbon to δ-gluconolactone (D-glucono-1,5-lactone), glucose 1-oxidase (GOx), and glucose 1-dehydrogenase (GDH). GOx and GDH are catalytically and phylogenetically closely related (Fig. 4), but differ in their preference for the electron acceptors employed in their oxidative half-reactions. While GOx preferentially reduces molecular oxygen to H_2_O_2_, GDH shows very low activity with O_2_ and utilizes a range of alternative electron acceptors.

GDH was not included in the original description of subfamily AA3_2 (Levasseur et al. 2013). Mori et al. (2011) showed for the first time that GOx and GDH cluster in distinct phylogenetic clades. A more detailed phylogenetic analysis of fungal GOx and GDH sequences performed by us revealed four distinct phylogenetic clades. Oxidases are only found in one of the four clades while the other three contain only dehydrogenases and uncharacterized enzymes. We propose to name these four clades GOx and GDH class-I to III. The GOx clade contains only ascomycete sequences including all glucose 1-oxidases characterized to date, GDH class-I comprises only ascomycete sequences including most of the currently characterized glucose 1-dehydrogenases, GDH class-II includes only ascomycete sequences that are completely uncharacterized to date, and GDH class-III comprises only basidiomycete sequences (with only one characterized member to date, see below). An interesting aspect of this analysis is the phylogenetic relationship between ascomycete GOx and basidiomycete GDH class-III, which is closer than that of GOx with other ascomycete GDH sequences. This, together with the longbranch length of GOx (0.8 substitutions per site) compared to GDH clades, indicates a high degree of specialization already from an early point in GOx/GDH evolution. In the past, enzymes of various organisms were often falsely annotated as GOx, leading to the belief that GOx is much more widely distributed among fungi than is actually the case. Later on, these falsely annotated GOx were often found to be GDH or POx instead. This wrong annotation can be easily explained by the high-sequence similarity in the case of GDH, and the utilization of the same substrates (glucose and O_2_) in the case of POx.

GOx (EC 1.1.3.4, β-D-glucose:oxygen 1-oxidoreductase) is a homo-dimeric glycoprotein with a non-covalently but tightly bound FAD cofactor. The first description of GOx from *A. niger* (*An*GOx) dates back to 1928 (Müller 1928). *An*GOx also was the first AA3 enzyme, of which the crystal structure was solved in 1993 (2.3 Å, PDB 1GAL (Hecht et al. 1993)). High-resolution structures of *An*GOx (1.9 Å, PDB 1CF3) and GOx of *Penicillium amagasakiense* (1.8 Å, PDB 1GPE) were published soon after (Wohlfahrt et al. 1999). GOx shows a very high preference for β-D-glucose, and hardly any other sugars are oxidized with significant catalytic efficiency. This specificity towards β-D-glucose stems from a highly specialized active-site architecture, characterized by the conserved residues Tyr, Thr or Ser, Arg, Asn, and the catalytic His pair (Y68, T110, R512, N514, H516, and H559 in *An*GOx 1CF3), resulting in the formation of hydrogen bonds to all five hydroxyl groups of β-D-glucose (Wohlfahrt et al. 1999; Yoshida et al. 2015). The C-6 hydroxyl group of β-D-glucose interacts with the conserved Thr or Ser, making GOx specifically selective over D-xylose (Sode et al. 2017). *An*GOx is the currently best-characterized GOx and is also most widely used in industrial applications. *An*GOx shows a catalytic efficiency of up to 1.5 × 10^6^ M^−1^ s^−1^ for D-glucose (Roth and Klinman 2003) and was once described as the “Ferrari” among flavin-dependent oxidases (Mattevi 2006).

Even though the first fungal glucose 1-dehydrogenase (GDH; EC 1.1.5.9 transferred in 2013 from 1.1.99.10, D-glucose:quinone 1-oxidoreductase) was already reported in 1937 in *Aspergillus* (Ogura and Nagahisa 1937), attention towards GDH developed only lately for its possible application in glucose biosensors independent of O_2_ levels. The first crystal structure of *Aspergillus flavus* GDH was solved recently (*Af*GDH; 1.78 Å, PDB 4YNT (Yoshida et al. 2015) and published together with a structure including the reaction product D-glucono-1,5-lactone (1.57 Å, PDB 4YNU), showing specific substrate-binding interactions. GDHs are found either as monomeric or homodimeric proteins. They are phylogenetically and structurally very closely related to GOx, showing both the same domain architecture and conserved catalytic residues. This conformational relatedness is also reflected in the root-mean-square deviation (rmsd) value for the backbone Cα atoms between *Af*GDH (4YNU) and *An*GOx (1CF3) of 1.31 Å. Together with structural features, GDH shares most of the active site composition and therefore the high-substrate specificity towards Glc with GOx, which typically is the preferred sugar substrate. Yet some GDHs show significant activity with D-xylose (Xyl), which can range from 2% relative activity of that with Glc to approximately equal activities for Glc and Xyl, depending on the source of the enzyme. This loss in specificity is attributed to a missing Thr or Ser, otherwise forming a hydrogen bond to the C-6 hydroxyl group of β-D-glucose (Sode et al. 2017). In contrast to GOx, GDH occurs both in ascomycota (GDH class-I and II) and basidiomycota (GDH class-III). To date only one class-III GDH from the basidiomycete *P. cinnabarinus* (*Pc*GDH) was characterized, which showed lower catalytic efficiency towards glucose than ascomycete GDH (Piumi et al. 2014). *Pc*GDH does not show the complete set of highly conserved residues responsible for glucose binding in both GDH and GOx (only Y64, H528, and H571 are conserved), yet D-glucose is by far the preferred substrate (Piumi et al. 2014). Detailed kinetic studies of GDHs are still scarce; the catalytic activity was determined for example for *Glomerella cingulata* class-I GDH (*Gc*GDH; 24.5 × 10^3^ M^−1^ s^−1^, D-glucose, and the ferrocenium ion as substrates; (Sygmund et al. 2011)) or for class-III *Pc*GDH (67 M^−1^ s^−1^ with D-glucose and DCIP as substrates; (Piumi et al. 2014)). GDH shows almost no reactivity with oxygen. Typical electron acceptors that are employed to re-oxidize its FAD include a range of differently substituted quinones or certain radicals such as phenoxy radicals.

### Subfamily AA3_2—pyranose dehydrogenase

Pyranose dehydrogenase (PDH; EC 1.1.99.29, pyranose:acceptor oxidoreductase) was first isolated and described from *Agaricus bisporus* (Volc et al. 1997). PDH is an extracellular, monomeric, glycosylated enzyme that is found in a rather restricted group of litter-degrading basidiomycetes belonging to the *Agaricaceae* and *Lycoperdaceae*, but not in white-rot wood-decaying fungi (Volc et al. 2001). The structure of PDH1 from *Leucoagaricus (Agaricus) meleagris* (*Lm*PDH1; 1.6 Å, PDB 4H7U, (Tan et al. 2013)) shows the typical two-domain architecture of the other AA3s, with a substrate- and a flavin-binding domain. In contrast to most other AA3s, FAD is covalently tethered to a His residue in the active site. The GMC family member structurally most similar to *Lm*PDH is *Pe*AAO with an rmsd value of 1.6 Å for 544 of 575 aligned Cα atom pairs, as well as *An*GOx with an rmsd value of 1.5 Å for 461 aligned Cα atom pairs (Tan et al. 2013), as also reflected in the close phylogenetic relationship of these three branches of subfamily AA3_2 (Fig. 1). The access to the active site of PDH is rather open and unobstructed, which is also reflected in the enzyme’s broad substrate reactivity, ranging from various mono- and oligosaccharides to even polysaccharides. Depending on the fungal source of the enzyme and the pyranose sugar substrate, PDH can catalyze the selective mono-oxidations at C-1, C-2, or C-3 of the sugar, or di-oxidations at C-2,3 or C-3,4 of the molecule, yielding the corresponding aldonolactones (C-1 oxidation) or (di)ketosugars [(di)dehydrosugars or aldos(di)uloses] (Peterbauer and Volc 2010). *Lm*PDH1, one of three isoforms of PDH from *L. meleagris*, is the biochemically and structurally best-studied representative of PDH*.* Based on the catalytic efficiencies, L-arabinose (Ara), D-glucose, and D-galactose (Gal) are the preferred sugar substrates of *Lm*PDH1 (62.1 × 10^3^ M^−1^ s^−1^, 57.5 × 10^3^ M^−1^ s^−1^ and 46.2 × 10^3^ M^−1^ s^−1^, respectively). While Ara and Gal are only oxidized at C-2, yielding the respective 2-keto sugars, Glc is oxidized both at C-2 and C-3, so that the final reaction product is 2,3-diketoglucose (Sygmund et al. 2008). As was shown by substrate turnover experiments and molecular dynamics simulations, oxidation at C-2 is preferred over C-3 oxidation (Graf et al. 2015). The reactivity of PDH with oxygen is negligible. PDH reduces a number of (complexed) metal ions (the ferrocenium ion, ferricyanide), variously substituted quinones (tetra-chloro-1,4-benzoquinone, 3,5,-di-t-butyl-1,2-benzoquinone) and the azino-bis-(3-ethylbenzthiazolin-6-sulfonic acid) (ABTS) cation radical, with the natural electron acceptor of PDH yet unknown (Kujawa et al. 2007). Three PDH-encoding genes were identified in *L. meleagris* (*pdh1*, *pdh2*, *pdh3*), coding for three PDH isoforms that share 75–92% amino acid similarity. *pdh2* and *pdh3* are essentially transcribed constitutively, yet at very low levels, whereas *pdh1* expression is up-regulated upon exhaustion of the carbon source and appears to be additionally regulated under conditions of oxygen limitation (Kittl et al. 2008). *A. bisporus* contains only a single *pdh* gene, which is expressed in the mycelium as well as the gills of the fruiting bodies, while expression is low in the stem and the hood of these fruiting bodies (Gonaus et al. 2016). In a comparison of the biochemical and kinetic properties of the three *Lm*PDH isoforms, it was shown that they possess comparable properties with respect to their electron donor sugar substrates (substrate specificity and kinetic properties). In contrast, their kinetic properties and relative activities differed significantly for model electron acceptor substrates studied, a radical (the ABTS cation radical), a quinone (benzoquinone), and a complexed iron ion (ferrocenium ion). Thus, a possible explanation for this multiplicity of PDH could be that in vivo the different PDH isoforms react preferentially with structurally different electron acceptors (Graf et al. 2017).

### Subfamily AA3_3—alcohol oxidase

Alcohol oxidase (AOx, sometimes referred to as methanol oxidase, MOx, based on the preferred substrate of the enzyme; EC 1.1.3.13, alcohol:oxygen oxidoreductase) was first described in 1965 in the basidiomycete *Polyporus obtusus* (renamed to *Spongipellis unicolor*) (Janssen et al. 1965). AOx catalyzes the FAD-dependent oxidation of lower, aliphatic primary alcohols (both saturated and unsaturated) to the corresponding aldehydes. It is typically not active on secondary alcohols (Gvozdev et al. 2012).

FAD-dependent AOx is a key enzyme of methylotrophic yeasts that can utilize methanol (as well as other short primary alcohols) as sole source of carbon and energy. It has been primarily studied in these methylotrophic yeasts including *Pichia*, *Candida*, or *Hansenula*, where it is located in the peroxisomes, to which it is targeted by a C-terminal signal sequence (Ozimek et al. 2005). AOx can comprise up to 30% of the total cellular protein in these organisms. The structure of the alcohol oxidase AOX1 from *Pichia pastoris* (*Pp*AOx) has been solved both by crystallography and X-ray diffraction (2.35 Å, PDB 5HSA, (Koch et al. 2016)) as well as by cryo-electron microscopy (3.4 Å, PDB 5I68 (Vonck et al. 2016)). *Pp*AOx is a homo-octameric protein, with each subunit carrying one non-covalently attached FAD. This FAD is modified and contains an arabityl rather than the canonical ribityl chain attached to the isoalloxazine moiety, which is formed by an autocatalytic reaction. It is thought that this FAD modification affects the reactivity of the enzyme with its alcohol substrates primarily by lowing the Michaelis constant for methanol. The extent, to which the flavin is modified, is inversely correlated to the methanol concentration in the growth medium and may vary from 5 to 95% (Ashin and Trotsenko 1998). The preferred substrate of *Pp*AOx as judged from the catalytic efficiency is methanol (9.58 × 10^3^ M^−1^ s^−1^); *k*_cat_·*K*_M_^−1^ values decrease significantly with longer chain length of the alcohol substrates (Koch et al. 2016).

AOx from basidiomycete and/or phytopathogenic fungi has received significantly less attention compared to their yeast counterparts. An extracellular AOx isolated from cultures of the brown-rot fungus *Gloeophyllum trabeum* (*Gt*AOx) displayed 50–53% sequence identity to other yeast and fungal AOxs, including *Pp*AOx (Daniel et al. 2007). Its C terminus is distinctly different from that of yeast AOx, and it also contains no typical N-terminal fungal signal sequence, yet immunofluorescence and TEM-immunogold labelling studies showed that *Gt*AOx was extracellularly localized, associated with hyphal cell walls as well as with extracellular slime. *Gt*AOx could not be shown in peroxisomes by TEM-immunogold labeling. The authors suggested that the C-terminal sequence of *Gt*AOx is responsible for protein translocation (Daniel et al. 2007). In accordance with yeast AOx, *Gt*AOx is a homo-octameric protein, with one non-modified and non-covalently attached FAD per subunit. Methanol is the preferred substrate of *Gt*AOx, which is turned over at a catalytic efficiency (6.78 × 10^3^ M^−1^ s^−1^) comparable to that of *Pp*AOx. Concomitantly, oxygen is reduced to hydrogen peroxide. In contrast to yeast AOx, which is believed to play a role in methanol metabolism and assimilation, it is thought that the role of *Gt*AOx (or AOx from other fungal brown-rotters) is to provide H_2_O_2_ for the fungal attack on lignocellulose. AOx is also found in the phytopathogenic basidiomycete *Moniliophthora perniciosa*, the causative agent of Witches’ broom disease in the cocoa tree (*Theobroma cacao*). AOx is also secreted in *M. perniciosa*, but it was proposed to play a role in the utilization of methanol derived from the demethylation of pectin rather than in the attack on the plant (de Oliveira et al. 2012).

### Subfamily AA3_4—pyranose oxidase

Pyranose oxidase (pyranose 2-oxidase, glucose 2-oxidase; POx, EC 1.1.3.10, pyranose:oxygen 2-oxidoreductase) is the most distantly related member of the AA3 family (Fig. 1) and does not show the otherwise high conservation of typical family motifs in the AA3 family. Therefore, it was rather late that POx was identified as a member of the GMC family (Albrecht and Lengauer 2003), even though POx was first described already in 1968 in the basidiomycete *Spongipellis unicolor* (*Polyporus obtusus*; (Janssen and Ruelius 1968). Crystal structures of *Trametes ochracea* (synonym *Trametes multicolor*) POx (*To*POx; 1.8 Å, PDB 1TT0; (Hallberg et al. 2004), *Peniophora* sp. POx (*Ps*POx, 2.35 Å, PDB 1TZL, (Bannwarth et al. 2004) and *P. chrysosporium* POx (*Pc*POx; 1.8 Å, PDB 4MIF; (Hassan et al. 2013)) show that POx is also the most diverse AA3 member with respect to structural features. In contrast to other AA3 family members, POx is a homotetrameric protein. Each of the two-domain subunits contains one active site with FAD as cofactor, covalently linked to a His. Access to the active site is restricted by a highly mobile active-site loop, which also restricts the activity of the enzyme to monosaccharide substrates. Access to the active sites is furthermore only possible through tunnels from the polypeptide surface to an internal large cavity formed by the four subunits. Each of the subunits carries a small extension termed “head domain” (Fig. 2) of an unknown function; it was speculated that this head domain is involved in oligomerization or in interactions with cell wall-polysaccharides or other proteins (Hallberg et al. 2004).

The preferred substrate for POx is D-glucose, but in contrast to GOx, POx can utilize both α- and β-D-glucose (the activity of GOx is restricted to α-D-glucose) as well as other monosaccharides including D-galactose, D-xylose, or D-glucono1,5-lactone at relevant rates (Leitner et al. 2001; Pisanelli et al. 2009) for its reductive half-reaction. POx catalyzes the oxidation of these aldopyranoses at position C-2, yielding the corresponding 2-ketoaldoses (2-dehydroaldoses or osones) as products. Some POx can additionally oxidize sugars at C-3, but this activity is typically much lower than oxidation on C-2 (Giffhorn 2000). Catalytic efficiencies for *Pc*POx and D-glucose are 98.9 × 10^3^ M^−1^ s^−1^ (oxygen/air as saturating substrate, (Pisanelli et al. 2009)). In the oxidative half-reaction, two electrons are transferred from the reduced FAD to O_2_ forming H_2_O_2_. The oxidative reaction of POx involves formation of a C4a-hydroperoxyflavin intermediate, which had previously not been detected in other flavin-dependent oxidases (Wongnate and Chaiyen 2013) but is the typically intermediate for monooxygenases. Apart from oxygen, POx can also utilize alternative electron acceptors including a number of (substituted) quinones and (complexed) metal ions. Interestingly, catalytic efficiencies for some of these electron acceptors are much higher than for molecular oxygen, indicating that these might in fact be the more relevant natural substrates than oxygen (*Pc*POx, 89.2 × 10^3^ M^−1^ s^−1^ and 11.9 × 10^6^ M^−1^ s^−1^ for oxygen and 1,4-benzoquinone, respectively; in each case D-glucose was the saturating substrate (Pisanelli et al. 2009)).

POx is found in a number of basidiomycetes, associated with membrane-bound vesicles and other membrane structures in the periplasmic space of the fungal hyphae (Daniel et al. 1992) but also extracellularly associated with polysaccharides. In a recent study, Mendes et al. showed that POx sequences can also be found in bacteria with sequence identities of 39–24% to fungal POx. The majority of these putative bacterial POx sequences occurred in the phylum of *Actinobacteria*. POx from *Arthrobacter siccitolerans* is the only characterized bacterial POx so far (Mendes et al. 2016), but POx activity was also proven in medium of the endophytic bacterium *Pantoea ananatis* when cultivated on rice straw (Ma et al. 2016a) suggesting that POx might be further spread throughout the bacterial domain.

### Biological functions of AA3 family members

**Fig. 5:**
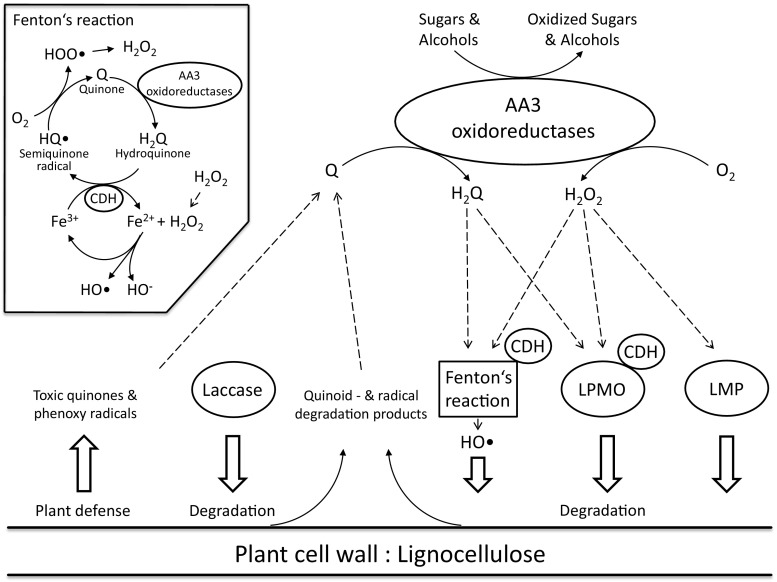
AA3 enzymes and lignocellulose degradation. This schematic presentation shows possible relevant interactions of different AA3 family oxidoreductases. H_2_O_2_, the reaction product of AA3 oxidases, can fuel lignin-modifying peroxidases (LMP), lytic polysaccharide monooxygenases (LPMO), and drives the Fenton reaction for attack on lignocellulose. Hydroquinones (H_2_Q), possible reaction products of AA3 dehydrogenase activity, may act as direct electron sources for LPMO and can take part in Fenton’s reaction cycle. Toxic compounds such as quinones (Q) and radicals emerging from lignin degradation or plant defense can be reduced by AA3 dehydrogenase activity. Cellobiose dehydrogenase (CDH) directly reduces LPMO and is also able to reduce Fe^3+^ ions for Fenton chemistry. The inset (top left) shows the full Fenton reaction including the reduction of Fe^3+^ by hydroquinone or CDH

Enzymes from the different AA families in the CAZy database do not directly act on polymeric constituents of lignocellulosic material (cellulose, various hemicelluloses, pectin, or lignin) but cooperate with other enzymes and thus help to depolymerise lignocellulose. The AA3 family, as defined by CAZy, consists of FAD-dependent GMC oxidoreductases (Levasseur et al. 2013) that support lignocellulose degradation by reducing low-molecular weight components such as oxygen and quinones as well as metal ions to some extent. The two main products that are formed are H_2_O_2_, resulting from the reduction of oxygen by the oxidases, and hydroquinones from the reduction of quinones by dehydrogenase activities (Fig. 5). Most of the proposed physiological functions that are attributed to AA3 family members can be deduced from these two reactions, and both products (peroxide and hydroquinones) support other enzymes or reactions important for the deconstruction of lignocellulosic material in different ways. Maybe one of the best known of these interactions is the supply of H_2_O_2_ by different oxidases to lignin-modifying peroxidases (LMPs; CAZy auxiliary activities family 2, AA2). Peroxidases employ peroxide as an electron acceptor in the classical peroxidase reaction cycle. Peroxidases in the resting ferric state react with H_2_O_2_ in a two-electron process to generate an intermediate known as compound I (an oxo-ferryl porphyrin cation radical complex), which then oxidizes two substrate molecules in two sequential single-electron abstractions, yielding radical products that are highly reactive. The first lignin-degrading enzymes evolved from a gene encoding a single generic peroxidase, and after gene duplication evolved further into a number of different lignin-degrading class-II peroxidases (Floudas et al. 2012), which play an important role in white-rot as well as in litter-degrading fungi (Lundell et al. 2014).

The role of H_2_O_2_ could even go beyond the activation of peroxidases. Recently, it was shown that LPMOs are activated by H_2_O_2_ (Bissaro et al. 2017), and hence various oxidases formed by LPMO-producing organisms might play an accessory role in the oxidative cleavage of polysaccharides. An AA9 LPMO was co-regulated and co-secreted with an AA3_2 enzyme in the white-rot fungus *P. cinnabarinus* (Miyauchi et al. 2017), and *Aspergillus nidulans* grown on starch secreted six AA3_2s (together with AA7 glyco-oligosaccharide oxidases) and starch-active AA13 LPMO. These various enzymes also showed similar secretion patterns (Nekiunaite et al. 2016), which could indicate an interaction between AA3 oxidases and various LPMOs. Several AA3 oxidases are known to be formed intracellularly, including for example GOx (Levasseur et al. 2013). Even these intracellular oxidases might contribute peroxide for extracellular reactions, as it was shown that certain members of the major intrinsic proteins, aquaporins, facilitate the diffusion of hydrogen peroxide through the membrane (Bienert and Chaumont 2014). Genes for these transmembrane proteins are found across all kingdoms of life but were very little studied in fungal organisms, and the function of aquaporins has not been elucidated unequivocally (Tanghe et al. 2006).

Hydrogen peroxide is also essential for a non-enzymatic attack on lignocellulosic material via highly reactive oxygen species formed by Fenton chemistry (Fe^2+^ + H_2_O_2_ + H^+^ − > Fe^3+^ + HO + H_2_O). This reactivity was first discovered as an attack on cellulose and lignocellulose when adding exogenous ferrous iron and hydrogen peroxide (Halliwell 1965; Koenigs 1974), but it is now understood that several enzyme activities contribute to the formation of these two compounds necessary for the Fenton reaction in an attack of brown-rot fungi on wood. Brown-rot fungi apparently lost the ancient manganese peroxidase gene and hence had to adopt a lifestyle different from white-rot organisms (Arantes and Goodell 2014; Lundell et al. 2014). Brown-rot fungi are thought to produce hydrogen peroxide primarily through the oxidation of methanol, which is released through demethylation from lignin substructures, by AA3_3 AOxs (Arantes and Goodell 2014). These are typically found in the genomes of brown-rot organisms, in contrast to the other oxidases implicated in peroxide formation in wood-degrading fungi, AAO and POx (Lundell et al. 2014). Recently, Fenton chemistry was also suggested to play a role in the degradation of rice straw by the endophytic bacterium *Pantoea ananatis* (Ma et al. 2016a). Strain *P. ananatis* Sd-1 was found to contain nine genes for AA enzymes, significantly more than the genomes of other *P. ananatis* isolates (Ma et al. 2016b). One of these genes codes for the AA3_4 enzyme POx, and it was suggested that in the bacterial system, POx provides the peroxide needed. Fe^3+^, which is found and mobilized in wood in concentrations sufficient to support Fenton chemistry, is thought to be reduced by small aromatic molecules such as 2,5-dimethoxyhydroquinone and related compounds released by brown-rot organisms (or resulting from the attack on lignin), which in turn are oxidized to the corresponding quinones. During the reduction of Fe^3+^ to Fe^2+^ by hydroquinones, semiquinone radicals are formed that can react with O_2_ to give HOO^**·**^ radicals. These can then dismutate to produce H_2_O_2_ or reduce another Fe^3+^ (Arantes and Goodell 2014). The hydroquinones driving this redox cycle can regenerate through reduction of the quinones by benzoquinone reductases (CAZy AA6), which are found in brown-rot fungi such as *Postia placenta* (Martinez et al. 2009). Alternatively, AA3 oxidoreductases (both dehydrogenases and oxidases) might employ these quinones as electron acceptors. GDH, PDH, and POx showed significant activity with various methoxylated quinones (Leitner et al. 2001; Pisanelli et al. 2009; Sygmund et al. 2011), and while PDH and POx are typically not found in brown-rot fungi, other AA3 enzymes might possess comparable catalytic properties. An alternative mechanism for direct iron reduction by an AA3 enzyme involves CDH. CDH is found in several brown-rot fungi such as *Fomitopsis pinicola*, *Gloeophyllum trabeum*, or *Serpula lacrymans*, yet not all brown-rotters form CDH (Lundell et al. 2014), so this does not seem to be a universal mechanism.

A quinone redox cycle comparable to that of brown-rot fungi for the provision of reduced iron was also proposed as one possibility to drive the reaction of LPMO. LPMO relies on the supply of electrons for its catalytic reaction, the oxidative cleavage of polysaccharides. These can be provided, among others, by CDH, as described above, directly by AA3 oxidoreductases (Garajova et al. 2016), or by different quinone compounds (Kracher et al. 2016). These quinone redox mediators can be regenerated and recycled by different AA3s thus fueling the activity of LPMO (Kracher et al. 2016).

The abovementioned reduction of quinones to hydroquinones by AA3s has recently been gaining more attention and could be important for several other functions, in addition to the ones mentioned before. This reduction can be part of a protective mechanism, by which fungi detoxify lignin degradation products or evade plant defense mechanisms. Plants produce toxic quinones during fungal degradation as a defense strategy. Some plants are also found to actively secrete phenoxy radicals to build up lignin as a response to fungal infection in order to physically block off fungal invasion—reduction of these radicals by fungal enzyme systems will counteract these plant defense reactions. A possible role of certain AA3s in these proposed mechanisms could be indicated by the expression of *Gc*GDH in the plant pathogen *Glomerella cingulata* (anamorph *Colletotrichum gloeosporioides)* in the presence of quinones rather than under carbon-limited conditions, as shown for CDH (Zamocky et al. 2006), POx (Vanden Wymelenberg et al. 2009) and PDH (Kittl et al. 2008). Furthermore, *Gc*GDH could reduce phenoxy radicals and thereby might ensure unhindered growth of the fungal hyphae upon plant infection (Sygmund et al. 2011). The phytopathogenic fungus *Ustilago maydis* secretes a considerable number of putative oxidoreductases when grown on maize bran (Couturier et al. 2012), and the most abundant of these oxidoreductases was identified as an aryl-alcohol oxidase (Couturier et al. 2016). It was suggested that the function of this AA3_2 enzyme is to provide H_2_O_2_ during plant invasion as this compound is thought to contribute to lesion formation and lesion expansion of plant cell walls in the infection mechanism of fungal pathogens (Govrin and Levine 2000).

These proposed functions of AA3 family members are supported by several recent studies on the secretome and/or the transcriptome of lignocellulose-degrading fungi when cultivated under different conditions. It was shown that *P. chrysosporium*, when grown on cellulose, lignin, or cellulose plus lignin supplemented cultures, secretes a number of AA3 enzymes in addition to numerous CAZymes involved in the decomposition of polysaccharides. The AA3_1 enzyme CDH was significantly up-regulated under cellulose conditions, whereas various AA3_2, AA3_3, and AA3_4 enzymes, including AAO, GOx-like enzymes, and AOx and POx were expressed and up-regulated in cultures with synthetic lignin as the main carbon source (Manavalan et al. 2011). Interestingly, POx was the most abundantly overexpressed protein under these growth conditions,and implies a function in lignin metabolism, which could be by providing H_2_O_2_ to lignin-attacking peroxidases but also by reducing quinones released from lignin to less toxic hydroquinones as mentioned above. Similarly, POx was up-regulated in *P. chrysosporium* when grown under carbon-limited conditions together with AAO and three other unidentified AA3_2 members (Vanden Wymelenberg et al. 2009).

Transcriptome and secretome analyses were used to compare wood decay by a white-rot and a brown-rot fungus, *P. chrysosporium* and *Postia placenta*, cultivated on aspen wood (Vanden Wymelenberg et al. 2010). While the former fungus employs a number of extracellular glycoside hydrolases to attack both cellulose and hemicellulose, *P. placenta* secreted an array of hemicellulases and fewer cellulases. Distinct differences were obvious with respect to the expression patterns of oxidoreductase-encoding genes. AOx was detected in both cultures, and it was concluded that methanol oxidation may be an important reaction to provide peroxide (for peroxidases or Fenton’s reaction) but also formaldehyde—formate dehydrogenases were up-regulated in both fungi concomitantly, so AOx might also be involved in the metabolism of methanol, which is released from lignin both in white and brown rot decay. In contrast to *P. chrysosporium*, *P. placenta* showed significant up-regulation of 1,4-benzoquinone reductase together with an unidentified FAD-dependent oxidoreductase (Ppl122772) and a glucose oxidase-like protein (Ppl128830) (Vanden Wymelenberg et al. 2010). The function of these two latter AA3 enzymes is, however, not known since the corresponding gene products have not been studied with respect to their substrate specificities and oxygen reactivity.

Multigenicity is a feature commonly found in fungal enzyme systems, especially in wood- and litter-degrading fungi, which often have to deal with harmful compounds derived from organic matter degradation, secondary metabolism of antagonists or human activities (Syed et al. 2014). This multigenicity is indicated by the presence of several paralogous genes in an organism as a result of gene duplication events (Taylor 2011), with the purpose of functional compensation upon deficiencies of one of its members (Salame et al. 2013). Moreover, gene duplication events are often followed by gene diversification, and this process is among the most important mechanisms leading to enzyme isoforms with new functionalities (Kilaru et al. 2006; Ramos et al. 2011). Explanations for the existence of multigenic AA family members have been given as functional redundancy, functional diversification including subtle differences in substrate specificity of individual enzymes, or fine-tuning of the gene regulation/expression in order to adapt fungal organisms to the diversity of their natural substrates (Lenfant et al. 2017; Levasseur et al. 2013). Examples for this fine-tuning and differences in expression of AA*3* genes as a response to different growth conditions and substrates can be found in the literature, e.g., different expression patterns of AA3 members when growing the plant-pathogenic white-rot fungus *Heterobasidion irregulare* on heartwood and reaction-zone wood of Norway spruce (Yakovlev et al. 2013), yet very few studies have looked at the biochemical properties and enzymological differences of individual AA3 family member isoforms from one single organism to better understand this multigenicity (Graf et al. 2017; Mathieu et al. 2016). Plant cell walls are very complex composite structures, and the multigenicity in the AA3 subfamilies might reflect necessities and adaptations to the degradation of these complex substrates.

Interestingly, this apparent need for multiple genes (and hence multiple enzyme activities) varies considerably between fungal species for the different subfamilies of AA3 enzymes. Ferreira et al. compared genes encoding different H_2_O_2_-producing GMC oxidoreductases (and hence AA3 enzymes) in ten genomes of Polyporales (Ferreira et al. 2015). This study again confirmed the multigenicity of different AA3 members to a varying extent—maximally one gene coding for AA3_1 CDH or AA3_4 POx were found in these ten fungal genomes, whereas up to eleven AAO (AA3_2) and six AOx genes (AA3_3) were identified in the genomes of *Bjerkandera adusta* and *Phlebia brevispora*, respectively.

To better understand the biological role of AA3 oxidoreductases and their multigenicity in fungal genomes, there is a clear need of more detailed biochemical characterization of various AA3 enzymes and extended studies on the occurrence and expression of AA3 genes in multiple fungal species. A remaining problem for such large-scale genome and lifestyle comparisons is the correct annotation of putative AA3 family enzymes. The challenge stems from the high-sequence identity throughout most of the AA3 family as well as the yet very limited set of biochemically characterized sequences/enzymes. Additionally, characterized enzymes are often restricted to a small sequence space, while a much more comprehensive characterization of the AA3 family would be necessary to ensure correct annotation. Lastly a recent study estimates the diversity of fungal species to be between 2.2 and 3.8 million (Hawksworth and Lucking 2017), with only 3–6% being identified to date. This suggests that currently we are only looking at a fraction of the genetic diversity in fungi and many more AA3 family—and other enzymes are still to be discovered.
